# Supplementary material for: Anti-Inflammatory and Antioxidant Properties of Tart Cherry Consumption in the Heart of Obese Rats
Source: Biology (Basel). 2022 Apr 23;11(5):646. doi: 10.3390/biology11050646 (PMC9138407; doi:10.3390/biology11050646)
Supplement: Supplementary file 1 [file biology-11-00646-s001.zip › biology-1652357-supplementary.pdf]

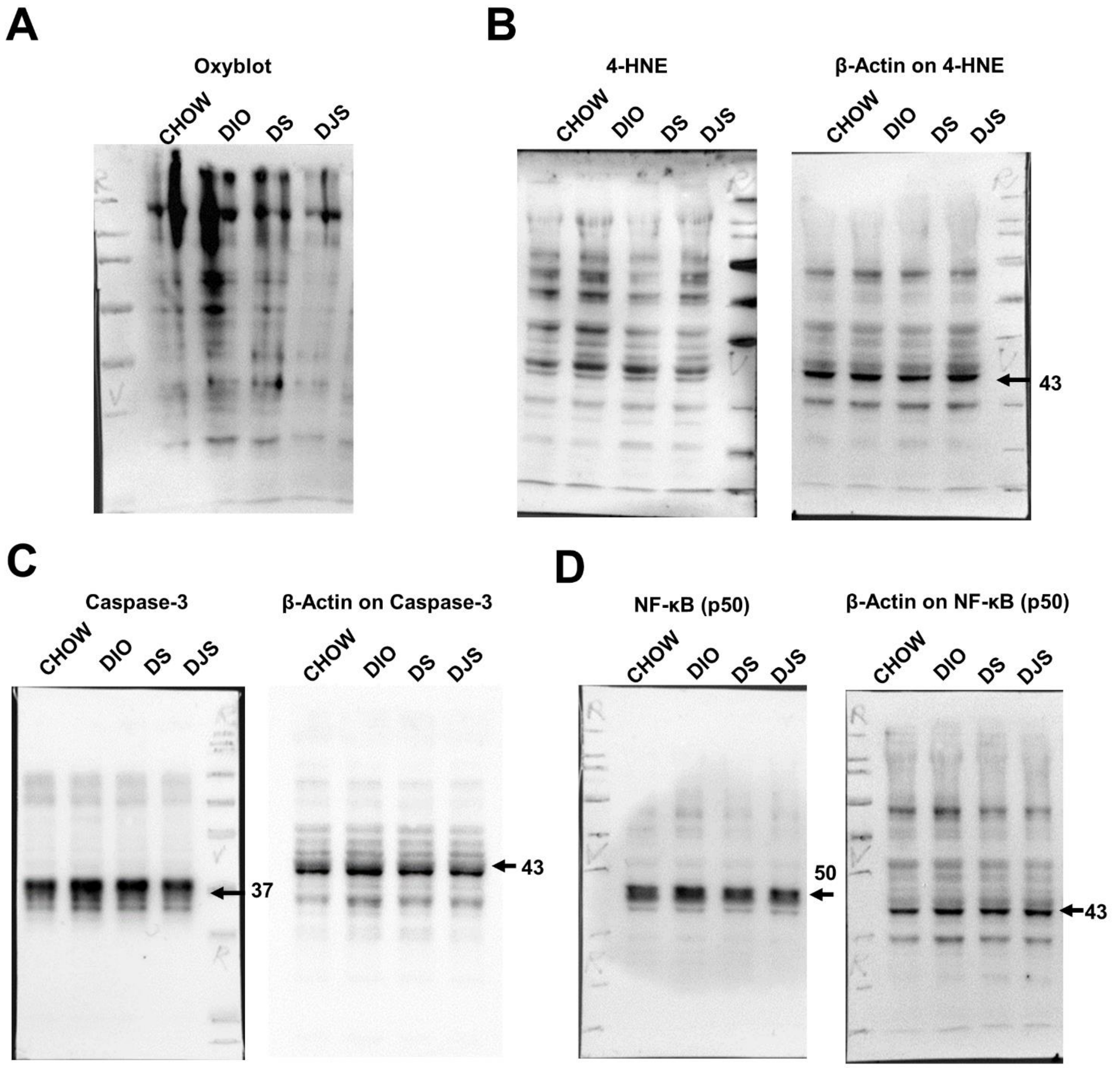

Supplementary Figure S1. Full-length WB images for figures 3 and 4: oxyblot (**A**), 4-Hydroxynonenal (4-HNE) (**B**), caspase-3 (**C**) and nuclear factor kappa-light-chain-enhancer of activated B cells subunit p50 (NF-κB p50) (**D**). All the WB were normalized to β-actin. Molecular weight of proteins in kilodaltons is indicated by the arrow at right. CHOW rats, fed with standard diet; DIO rats, fed with high-fat diet; DS, DIO rats supplemented with tart cherry seeds; DJS, DS rats supplemented with tart cherry juice.

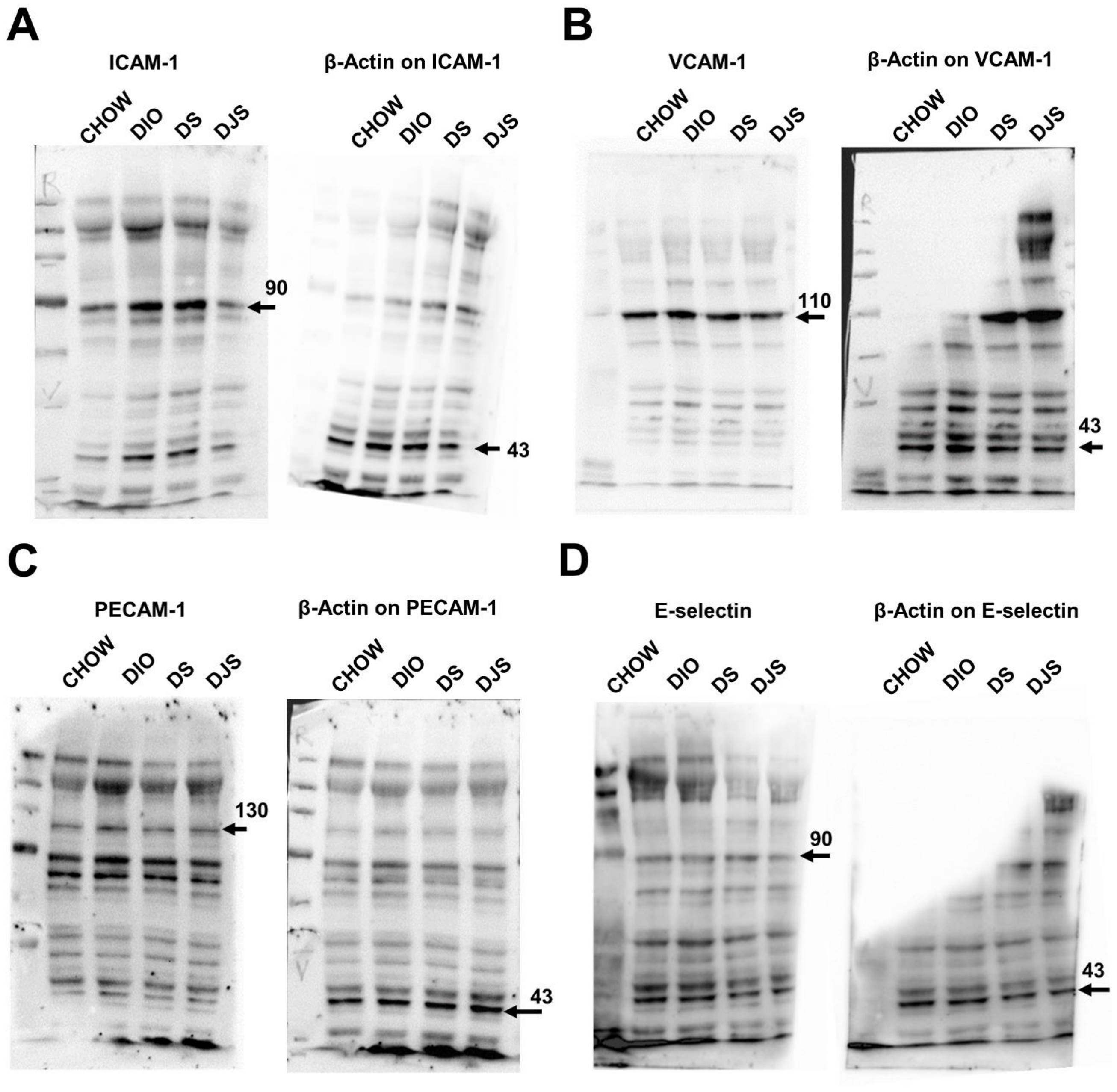

Supplementary Figure S2. Full-length WB images for figure 5: intracellular adhesion molecule-1 (ICAM-1) (**A**), vascular cell adhesion molecule-1 (VCAM-1) (**B**), platelet endothelial cell adhesion molecule-1 (PECAM-1) (**C**) and E-selectin (**D**). All the WB were normalized to β-actin. Molecular weight of proteins in kilodaltons is indicated by the arrow at right. CHOW rats, fed with standard diet; DIO rats, fed with high-fat diet; DS, DIO rats supplemented with tart cherry seeds; DJS, DS rats supplemented with tart cherry juice.

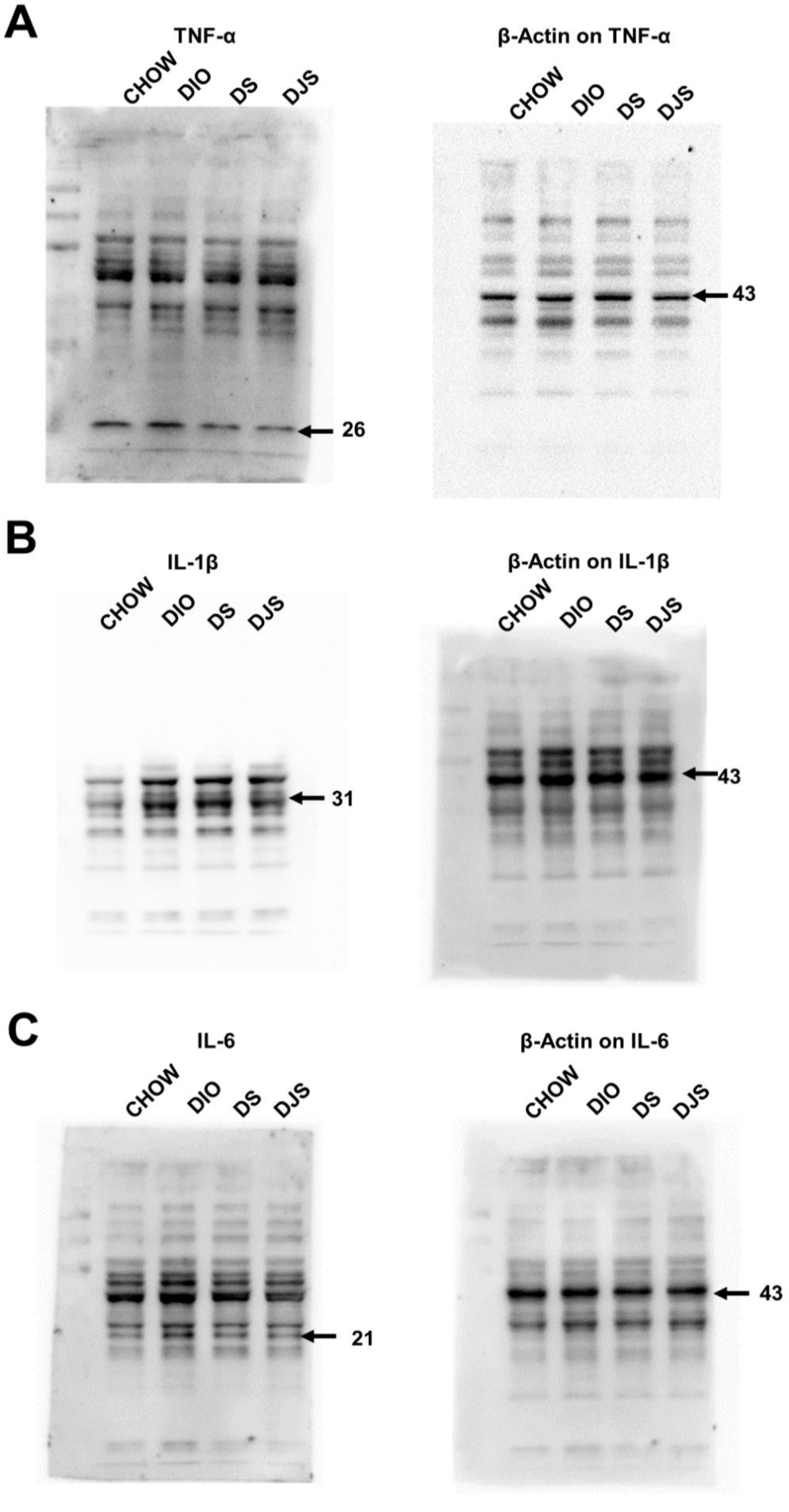

Supplementary Figure S3. Full-length WB images for figure 7: tumor necrosis factor-α (TNF-α) (**A**), interleukin-1β (IL-1β) (**B**) and interleukin-6 (IL-6) (**C**). All the WB were normalized to β-actin. Molecular weight of proteins in kilodaltons is indicated by the arrow at right. CHOW rats, fed with standard diet; DIO rats, fed with high-fat diet; DS, DIO rats supplemented with tart cherry seeds; DJS, DS rats supplemented with tart cherry juice.
